# Kidney Function and Size in Children With Down Syndrome: A Cross‐Sectional Study

**DOI:** 10.1111/nep.70007

**Published:** 2025-02-19

**Authors:** Flavia Padoan, Rossella Stifano, Milena Brugnara, Matteo Guarnaroli, Michele Piazza, Silvana Lauriola, Giorgio Piacentini, Angelo Pietrobelli, Luca Pecoraro

**Affiliations:** ^1^ Pediatric Unit, Department of Surgical Sciences, Dentistry, Gynecology and Pediatrics University of Verona Verona Italy

**Keywords:** down syndrome, eGFR, renal function, renal hypoplasia

## Abstract

Down syndrome (DS) is associated with a high prevalence of congenital heart, gastrointestinal, and endocrine anomalies, as well as a heightened risk for kidney and urinary tract abnormalities. The renal anomalies occur in up to 3.2% of DS cases at birth—four to five times higher than in the general population. Despite this, current DS management guidelines lack routine kidney evaluations, even though risk factors like neonatal acute kidney injury, renal hypoplasia and obesity may predispose DS children to chronic kidney disease (CKD). In a cross‐sectional study, we analysed kidney size and function in 54 DS children. Results revealed that 25% of patients exhibited renal hypoplasia, 26% had an estimated glomerular filtration rate (eGFR) below 90 mL/min/1.73 m^2^ Among adolescents, 55.5% showed eGFR values below 90 mL/min/1.73 m^2^ Additionally, 29.6% of the cohorts were overweight and 7.4% obese. There is a need for early kidney assessments in DS patients to detect initial renal decline and underscore the importance of close monitoring, particularly in adolescents. Further studies are needed to identify specific prognostic factors to better assess CKD risk in DS children, and limited research exists on renal replacement therapies for this population.

## 1 Introduction

Down syndrome (DS) is the most common chromosomal disorder, affecting approximately 1 in 700 live births globally. This syndrome exhibits a range of medical and developmental challenges, including an increased prevalence of congenital heart defects and gastrointestinal and various endocrinological abnormalities. Kidney and urinary tract anomalies constitute the fourth most common major congenital disability in DS, with prevalence at birth varying up to 3.2%, four to five‐fold higher than the general population [1]. Current guidelines do not yet include kidney evaluation in routine workups for DS. Nevertheless, these children present many risk factors regarding the development of kidney diseases, including neonatal acute kidney injury, small for gestational age, renal hypoplasia and childhood obesity [2, 3, 4]. As described in autopsy‐based studies, kidneys in trisomy 21 patients tend to be smaller than the general population [3]. This could be attributed to elevated endostatin levels, an angiogenesis inhibitor encoded on chromosome 21 [5]. Renal hypoplasia is determined by kidney length below two standard deviations (3rd percentile) of that of age‐matched normal individuals [6]. Early‐life ultrasound screenings with the assessment of renal size could be useful in detecting many of these anomalies. Timely diagnosis and management of these conditions are critical in preventing potential complications. Kidney and urinary tract anomalies observed in down children, such as renal hypoplasia, constitute an important clinical consideration due to their potential increased risk for chronic kidney disease (CKD) development [7]. Congenital anomalies of the kidney and urinary tract (CAKUT), such as renal hypoplasia, represent the leading cause of CKD in children [7]. Moreover, kidney hypoplasia/aplasia and dysplasia represent the second most common primary diagnosis in children with end‐stage renal disease, accounting for 14.2% of the patients who undergo dialysis in paediatric age [8, 9]. Studies have shown that children with DS present an overt decrease in kidney function as compared with healthy controls [3, 10]. The KDIGO 2012 guidelines define CKD as abnormalities of kidney structure or function with health implications and require one of two criteria documented or inferred for > 3 months: either GFR < 60 mL/min/1.73 m^2^ or markers of kidney damage (albuminuria > 30 mg/day, urine sediment abnormalities, electrolyte and other abnormalities due to tubular disorders, abnormalities detected by histology or imaging, history of kidney transplantation) [11]. CKD is classified into six eGFR categories. In the absence of kidney damage, stages G1 (≥ 90 mL/min/1.73 m^2^) and G2 (between 60 and 89 mL/min/1.73 m^2^) cannot be considered CKD; however, stage G3 (between 59 and 30 mL/min/1.73 m^2^) and higher are always considered CKD, as only the GFR criterion is relevant for the CKD diagnosis [11]. While CKD is a recognised complication in individuals with DS, large‐scale studies precisely measuring CKD stage prevalence in children with DS are lacking, and long‐term studies assessing CKD progression are needed to provide more precise data.

## 2 Methods

We performed a cross‐sectional study to analyse kidney size and function in a cohort of children recruited between September 2022 and June 2023 during the outpatient visits for DS at the Paediatrics Unit in Mother and Child University Hospital in Verona, Italy. The parents or legal guardians signed the informed consent. This study was approved by the Ethics Commission for Clinical Trials of the Provinces of Verona and Rovigo (DONUT STUDY, code number 66508, 7 December 2020; amendment, code number 45686, 25 July 2022). The patients underwent blood tests and a whole abdominal ultrasound during the visit. The blood tests included a complete blood count and kidney function indices. The following data have been collected: demographic and clinical characteristics of the patients, kidney function indices and instrumental investigations performed during the diagnostic workup. Continuous data were described using median and interquartile range (IQR), and categorical data were shown as percentages.

## 3 Results

Fifty‐four children (28 males and 26 females) were selected in the context of the follow‐up for Down syndrome. The median age at the time of evaluation was 87 months. Most children presented cardiac, endocrinological, gastrointestinal and renal comorbidities. Notably, the prevalence of renal comorbidities was higher in our population compared with data from the literature (9% vs. 3.2%) [12]. All children underwent a whole abdominal ultrasound, which revealed renal hypoplasia in 25% of cases. Additionally, creatinine levels were measured, and renal function was calculated using the Schwartz formula. Twenty six percent of our patients had an eGFR below 89 mL/min/1.73 m^2^. Specifically, 9.3% of our children have eGFR < 75 mL/min/1.73 m^2^ (Table 1). This data are in line with other studies [3]. Limiting to the low sample size, it is important to emphasise that 5 of the 9 adolescents (55.5%) presented an eGFR lower than 90 mL/min/1.73 m^2^. This result is greater than what was found in the general paediatric population [13], and it is consistent with the results of Ardissino et al. [14]. Moreover, 29.6% of our children are overweight and 7.4% are obese. The extra weight forces the kidneys to work harder and filter above the normal level. Over time, this additional work increases the risk of CKD. In the context of this cross‐sectional study, no association was found between kidney size, BMI, and the level of GFR in this subset of patients. In general, no statistical associations involving these parameters were found in this cross‐sectional study, probably due to the low sample size.

**TABLE 1:** Baseline patients' characteristics and results.

	*n* = 54
Age, months	87 (66–123)
Gender, m (%)	28 (51.9)
BMI, kg/m^2^	17.9 (16.1–20.4)
Normal weight, *n* (%)	34 (63.0)
Overweight, *n* (%)	16 (29.6)
Obese, *n* (%)	4 (7.4)
Patients with comorbidities, *n* (%)	48 (89)
Cardiovascular, *n* (%)	37 (69)
Gastrointestinal, *n* (%)	2 (4)
Endocrinological, *n* (%)	10 (19)
Nephro‐urological, *n* (%)	5 (9)
Kidney size, cm	
Right	7.0 (6.4–7.7)
Left	7.3 (6.0–8.2)
Kidney size < 3rd percentile, *n* (%)	14 (25)
Creatinine, mg/dL	0.47 (0.38–0.57)^a^
eGFR, mL/min/1.73 m^2^	104 (88.0–120.0)^a^
≥ 90 mL/min/1.73 m^2^	37 (68.5)
between 60 and 89 mL/min/1.73 m^2^	13 (24.1)
≤ 59 mL/min/1.73 m^2^	1 (1.9)

*Note:* Unless otherwise specified, all data are shown as median and interquartile range (IQR).

^a^ Not determined for two patients.

## 4 Discussion

Our study demonstrates that 25% of paediatric patients with DS exhibit renal hypoplasia, and additionally, 26% of them present an eGFR below 90 mL/min/1.73 m^2^. Focusing on adolescents, 55.5% show an eGFR below 90 mL/min/1.73 m^2^. This finding is particularly noteworthy, as adolescence represents a critical period for paediatric patients with CKD [15], and this risk is even higher in those with DS. The limited renal functional reserve in these patients may lead to a decline in kidney function during this stage of life [16].

Moreover, considering the increased life expectancy of individuals with DS, it is significant to recognise CKD as a potential cause of mortality in this population. Currently, only a limited number of studies have examined renal replacement therapies and kidney transplantation in this group, describing the successful possibility of both replacement therapies in DS patients [17].

This study has some limitations. First, the low sample size. Second, data were collected in a single follow‐up program available for down syndrome patients at Verona University Hospital. To gain a more comprehensive understanding of creatinine levels and eGFR in children with DS who also present with renal hypoplasia, further studies involving larger population samples are required.

An early assessment of kidney function and size in children with DS appears to be essential for detecting the initial signs of renal function decline. It is crucial to determine optimal follow‐up intervals for paediatric DS patients, using clinical, laboratory and ultrasound evaluations to identify those needing closer monitoring, especially adolescents. These findings also guide whether monitoring should focus only on those with renal hypoplasia or extend to all DS patients, with tailored schedules for different risk groups.

The finding that 55.5% of DS adolescents have eGFR values below the normal range suggests that this group may benefit from closer monitoring and a more frequently scheduled follow‐up.

Further studies are necessary in order to identify more specific prognostic factors that could help distinguish different risk classes for kidney damage in DS children.

## Conflicts of Interest

The authors declare no conflicts of interest.

## Data Availability

The data that support the findings of this study are available from the corresponding author upon reasonable request.
